# Spray-Dried Phenolic Compounds from Olive Mill Waste Water as Animal Feed Supplement: Impact on the Aromatic Profile of “Caciotta Cheese”

**DOI:** 10.3390/molecules30193991

**Published:** 2025-10-05

**Authors:** Giulia Francesca Cifuni, Pasquale Caparra, Enzo Perri, Cinzia Benincasa, Giuseppe Morone, Salvatore Claps

**Affiliations:** 1 CREA Research Centre for Animal Production and Aquaculture, 00015 Rome, Italy; giuseppe.morone@crea.gov.it (G.M.); salvatore.claps@crea.gov.it (S.C.); 2 Division of Animal Production, Department of Agriculture, Mediterranean University of Reggio Calabria, 89124 Reggio Calabria, Italy; pasquale.caparra@unirc.it; 3 CREA Research Centre for Olive, Fruit and Citrus Crops, 87036 Rende, Italy; enzo.perri@crea.gov.it (E.P.); cinzia.benincasa@crea.gov.it (C.B.)

**Keywords:** phenolic compounds, waste valorization, cheese aroma profile, E-nose analysis, key volatile compounds

## Abstract

This study evaluated the effect of dietary supplementation with different levels of spray-dried phenolic compounds, extracted from olive mill wastewater, on the volatile compound profile of Caciotta cheese produced from cow’s milk. Thirty dairy cows were divided into three groups and fed diets containing 0% (C), 0.1% (T0.1), and 0.2% (T0.2) polyphenols on a dry matter basis. Milk from each group was used in three cheesemaking sessions, and 27 cheese samples ripened for 21 days were analyzed. Volatile compounds were extracted using solid phase microextraction (SPME) coupled with mass spectrometry, while the odour fingerprint was assessed using an electronic nose (PEN3). Principal Component Analysis (PCA) revealed a clear separation among groups, indicating distinct aromatic profiles associated with dietary polyphenol levels. In summary, incorporating by-products from olive mill wastewater into the diets of dairy cows can significantly affect the aroma of cheese. This approach represents a sustainable and innovative strategy that promotes waste valorization, reduces environmental impact, and supports circular economy principles by transforming a pollutant into a valuable additive.

## 1. Introduction

Agro-industrial processing for both food and non-food production generates numerous by-products and residues rich in bioactive compounds, particularly polyphenols [1]. In some instances, these by-products contain higher concentrations of bioactive compounds than the original raw materials. A notable example is olive mill wastewater (OMWW), a major by-product of olive oil extraction [2].

OMWW is a liquid effluent resulting from the water used during various stages of oil production, as well as the vegetable water naturally present in olives. It contains tannins, lignins, long-chain fatty acids, reducing sugars, proteins, and phenolic compounds, many of which are phytotoxic and harmful to microorganisms and plants [3]. Nevertheless, its high polyphenol content represents a valuable resource that can be recovered and reused for sustainable applications.

In recent years, increasing attention has been devoted to the recovery of phenolic compounds from OMWW, with promising applications in the food, feed, cosmetic, and pharmaceutical sectors. This transformation of a problematic waste into an economically and environmentally valuable resource has been widely documented [4]. Innovative membrane technologies, such as microfiltration, ultrafiltration, and nanofiltration, and spray-drying technologies [5], have been developed to extract phenolic compounds from olive oil by-products. Among the principal phenolic compounds identified in spray-dried olive mill wastewater oleuropein derivatives are the most abundant (54.67%), followed by tyrosol (17.03%), hydroxytyrosol (12.35%), verbascoside (5.83%), and hydroxytyryloleate (4.70%) [6]. The growing interest in phenolic compounds derived from olive oil by-product wastes is linked to their diverse and potent biological activities, including antioxidant, antiatherogenic, antihepatotoxic, hypoglycemic, anti-inflammatory, antitumor, antiviral, and immunomodulatory effects [7,8]. Oleuropein, hydroxytyrosol, tyrosol, and caffeic acid are recognized as effective scavengers of reactive oxygen and nitrogen species (ROS and RNS) [9]. Hydroxytyrosol, in particular, exhibits anti-inflammatory effects by attenuating pro-inflammatory signalling pathways in human monocytes [10].

In animal nutrition, the use of unconventional agro-industrial by-products rich in polyphenols as dietary supplements has demonstrated beneficial effects on animal performance, including improved protein utilization [11], oxidative status, and enhanced quality and stability of derived products [12,13].

Olive oil by-products, which are rich in antioxidants, may be incorporated into animal feed to reduce oxidative stress in livestock and improve the antioxidant status and oxidative stability of their meat and milk. Research indicates that these by-products enhance quality parameters, including shelf life, in meat from chickens [14], rabbits [15], beef [16], and pork [17,18], as well as in cheeses produced from cow milk [19].

Moreover, the inclusion of agricultural by-products, particularly those containing tannins and phenolic compounds, in ruminant diets has been shown to improve feed efficiency, reduce methane emissions, and lower feed costs. These outcomes contribute to environmental sustainability and support the development of circular economy models in livestock production systems [20].

Flavour compounds are critical determinants of product quality and consumer acceptance. The volatile compounds responsible for the characteristic aroma of cheese are primarily generated through lipolytic and proteolytic pathways, as well as through the metabolism of lactose, lactate, and citrate. These biochemical reactions produce volatile organic compounds (VOCs), such as aldehydes, alcohols, ketones, and esters, which contribute to the distinctive flavour of cheese and serve as indicators of ripening. The relative abundance of each volatile compound depends on the biochemical processes occurring during ripening, which are mediated by endogenous enzymes and microbial activity. These processes are strongly influenced by bioactive compounds ingested through the diet and transferred into milk via the mammary gland [21].

The inclusion of specific phenolic compounds from olive by-products, such as olive leaves, pomace, and wastewater, can significantly affect the chemical and nutraceutical properties of milk and cheese, with notable changes in the aromatic profile of cheeses [22,23]. It is well established that dietary modifications can induce changes in the chemical composition of milk, which are subsequently reflected in cheese. These changes provide different substrates for the metabolic activity of starter and non-starter bacteria, as well as for endogenous lipolytic and proteolytic enzymes. Consequently, the volatile and sensory characteristics of cheeses are largely determined by both the technological approach and the initial chemical composition of the raw milk [24].

This study aimed to assess the effect of dietary supplementation with varying levels of spray-dried phenolic compounds, extracted from olive mill wastewater, on the volatile compound profile of cheese made from dairy cow milk.

## 2. Results and Discussion

### 2.1. Chemical Composition of Cheese

Dietary supplementation with phenolic compounds did not alter the chemical composition of cheese (Table 1). These findings align with previous research investigating the impact of phenol supplementation, particularly from sources such as olive by-products [22], winemaking industry residues [23], on cheese composition and water activity, which are key factors influencing microbial metabolism and aroma development.

### 2.2. Spray-Dried Olive Mill Wastewater Phenols Composition

The application of low-temperature spray-drying technology enabled the production of a phenol-rich product with high biological value. As previously reported [5,6], the principal phenolic compounds identified in spray-dried olive mill wastewater are derivatives of oleuropein, which represent the most abundant fraction (54.67%). These are followed by tyrosol (17.03%), hydroxytyrosol (12.35%), verbascoside (5.83%), and hydroxytyryloleate (4.70%).

### 2.3. Volatile Profiles of Caciotta Cheese

**Figure 1:**
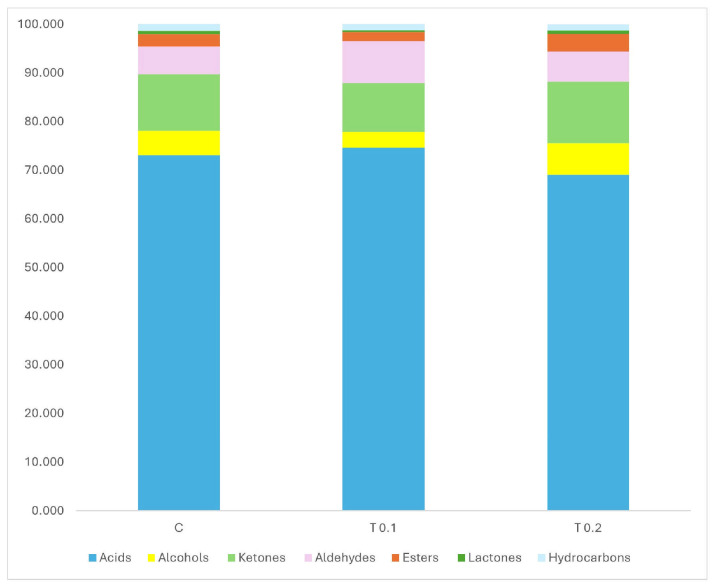
Effect of dietary supplementation with polyphenols on the classes of volatile compounds (%) in Caciotta cheese. C = Control diet, T0.1 = diet with polyphenols at 0.1% of dry matter, T0.2 diet with polyphenols at 0.2% of dry matter.

Dietary integration with different levels of biophenols, recovered by spray-drying technologies from olive mill wastewater, into the cow’s diet led to significant changes in the volatile profile of Caciotta cheese after 21 days of ripening (Figure 1).

A total of 45 compounds, including ketones, alcohols, aldehydes, esters, lactones, hydrocarbons, and fatty acids, were identified. Cheese samples from the T0.2 group showed a significant increase in the proportions of alcohol and ester (*p* < 0.0001) compared to the control (C) and T0.1 samples.

Conversely, cheese samples from the T0.1 group exhibited significantly higher aldehydes (*p* < 0.01) content and lower levels of lactones (*p* < 0.01) compared to the C and T0.2 samples (Figure 1). The most abundant compounds identified in all experimental cheeses were carboxylic acids, indicating that lipolysis was the primary process during cheese ripening [24]; however, it is also important to underline that the carboxylic acids can, in turn, represent the substrate of multiple oxidative mechanisms, giving origin to aldehydes, ketones, lactones and esters [25].

Dietary integration of polyphenols modified free fatty acids (FFAs), and it was particularly evident for butanoic, nonanoic, decanoic, dodecanoic, tetradecanoic and hexadecenoic acids (Table 2). Short-chain free fatty acids are linked to strong, sweaty, cheesy, and lipolytic aromas. Due to their lower odour thresholds, they primarily contribute to the aroma of dairy products. In contrast, decanoic and dodecanoic acids have higher odour thresholds and contribute less to the aromatic profile of dairy products [19].

Ketones were the second most abundant group of compounds in all experimental products. Acetoin (3-hydroxy-2-butanone) was present in lower proportions in T 0.2 cheese samples compared to those from T 0.1 and C group (*p* < 0.001). The inclusion level of up to 0.2% (of dry matter basis) of spray-dried polyphenols, from olive oil mill wastewater, into cows’ diet was effective in increasing 2,3 butanedione (diacetyl) and 2,3 butanediol levels in cheese samples (*p* < 0.001, respectively). Diacetyl, acetoin, and 2,3-butanediol are key aroma compounds contributing to the buttery and nut-like flavours characteristic of immature soft cheeses [26]. These compounds are metabolic by-products formed during the fermentation of lactose, primarily through glycolysis by both starter and non-starter lactic acid bacteria. More specifically, their biosynthesis is closely linked to citrate metabolism, a pathway active in certain lactic acid bacteria such as *Lactococcus lactis* and *Leuconostoc* species [27]. The production of 2,3-butanedione (diacetyl) is primarily attributed to the metabolic activity of lactic acid bacteria, notably *Lactococcus lactis* subsp. *lactis biovar diacetylactis* and *Leuconostoc* species [28]. These bacteria are known for their role in flavour development in fermented foods due to their ability to produce volatile compounds. Our results suggest that dietary supplementation with polyphenols may stimulate the growth or metabolic activity of certain beneficial bacteria, thereby enhancing the production of odour-active compounds such as diacetyl. This observation aligns with findings by Barbaccia et al. [29], who reported that phenolic extracts can positively influence the growth of specific Lactobacillus species. Ketones, commonly found in dairy products, are primarily derived from the β-oxidation of fatty acids [30]. These volatile compounds possess distinctive odours and are detectable even at low concentrations, contributing significantly to the sensory profile of cheese [27]. In our study, 2-pentanone and propanone were found in significantly higher concentrations in the polyphenol-supplemented groups (T0.1 and T0.2) compared to the control group (*p* < 0.001), suggesting a stimulatory effect of supplementation on ketone production.

This observation is consistent with findings by Innosa et al. [31], who reported that enriching goats’ diets with olive leaves led to increased levels of 2-pentanone in ripened goat cheeses. Interestingly, the T0.1 group also exhibited a significantly higher concentration of 2-undecanone (*p* < 0.001) and a lower percentage of 2-nonanone (*p* < 0.01) compared to other experimental groups. Methyl ketones in dairy products primarily originate from the microbial degradation of acyl lipids via the β-oxidation pathway [28]. Among these, propanone can be derived from butanoic acid, and may also be produced in mammalian glands, subsequently being transferred into milk [32].

These compounds are recognized for their unique aromatic characteristics, as 2-nonanone imparts fruity, spicy, and musty notes that are typical of most dairy products [26].

Dietary inclusion of spray-dried polyphenols from olive oil mill wastewater at levels up to 0.2% (dry matter basis) in cows’ diets resulted in significantly increased concentrations of ethyl hexanoate and ethyl octanoate in cheese (*p* < 0.001). Conversely, hexanoic methyl ester (*p* < 0.01) and octanoic methyl ester were more abundant in cheese samples from the T0.1 group compared to other experimental groups. This is an unexpected finding for immature cheese, as ester compounds typically emerge later in the lipolytic process.

Similar results were reported by Bennato et al. [33], who observed ester formation in soft cheese produced from milk of cows supplemented with licorice root. The ester content in cheese is governed by the balance between ester synthesis and fat hydrolysis, with esterases from lactic acid bacteria (LAB) playing a dual role: hydrolyzing milk fat glycerides to release free fatty acids (FFAs) and synthesizing esters from glycerides and alcohols via transferase reactions [34].

The presence of esters in immature cheese may be attributed to enhanced esterase activity of LAB, potentially stimulated by polyphenols in the cows’ diet, which have been shown to affect the growth of Lactobacillus species [28]. Notably, ethyl-hexanoate and ethyl octanoate contribute to sweet, fruity, and floral notes in cheese [35] and have low perception thresholds [28]. These esters also play a role in enhancing cheese flavour by mitigating the sharpness of fatty acids and reducing bitterness from amines [26].

Two lactones, δ-nonalactone and δ-decalactone, were identified in all experimental cheese samples. Notably, δ-decalactone was found at higher levels in the T0.2 group (*p* < 0.0001), while δ-nonalactone was lower (*p* < 0.001) in the T0.1 group. Lactones are cyclic esters formed through the esterification of hydroxy fatty acids [28] and are recognized for their fruity aroma and low perception thresholds. They are typically associated with peach and coconut-like odours [26] and contribute significantly to the sensory profile of cheese.

The dietary integration of polyphenols at a low level (0.1% of dry matter) increases nonanal (*p* ≤ 0.001), 2-nonenal (*p* ≤ 0.001) contents in cheeses, while the higher contents of 2-decenal (*p* < 0.0001) and pentadecanal (*p* < 0.001) occur in cheese made from the supplemented T0.2 group compared to other ones. Finally, a higher content of pentanal (*p* < 0.05) and dodecanal (*p* < 0.001) was found in cheese from the control group compared to the supplemented groups.

Aldehydes are transitory compounds in cheese, often rapidly reduced to primary alcohols or oxidized to corresponding acids. Straight-chain aldehydes such as n-butanal, n-pentanal, n-hexanal, and n-nonanal are typically formed via β-oxidation of unsaturated fatty acids [26] and at low concentrations, they impart green grass and herbaceous green grass and herbaceous odours. Further, the unsaturated form (E)-nonenal confers a pleasant green flavour to cheese.

Dietary supplementation with higher amounts of polyphenol leads to an increase in the level of ethanol (*p* < 0.05), 2–3 butanediol (*p*< 0.01) and 1-octadecanol (*p* < 0.01) in cheese. In contrast, cheeses from the control group showed significantly higher levels of 1-butanol (*p* < 0.0001), 1-octanol (*p* < 0.05), and 1-nonanol (*p* < 0.05).

Many metabolic pathways are involved in the synthesis of the alcohols found in cheese: lactose metabolism, methyl ketone reduction, amino acid metabolism, as well as degradation of linoleic and linolenic acids [36].

Ethanol is produced by the fermentation of lactose or citrate, or from alanine catabolism. 2,3-Butanediol can be generated from glycolysis or citrate metabolism of several LABs through acetoin reduction [37].

Our findings indicate that both ethanol and 2,3-butanediol, which are produced through similar metabolic pathways [37], exhibit comparable trends in cheeses made from cow’s milk that have been enriched with higher levels of phenolic compounds. This suggests that incorporating polyphenols into the cows’ diet may enhance the growth of starter lactic acid bacteria, which are responsible for lactose metabolism.

Consequently, this leads to a marked increase in the production of both ethanol and 2,3-butanediol, highlighting the importance of animal diet in cheese flavour development.

### 2.4. Influence of Dietary Polyphenols on Odour Impact Ratio Values (OIRs) and Identification of Key Volatile Compounds in Caciotta Cheese

The calculation of odour impact ratio values (OIR) is commonly used to identify the key odour-active compounds in food [38].

Typically, consumers evaluate the acceptability of food based on its aroma and flavour. In cheese, the odour activity values (OIRs) of volatile organic compounds are a crucial sensory characteristic that influences the quality of the final product.

The levels of OIRs can reflect the contributions of VOCs to the cheese’s characteristic flavour. In this study, the OIRs of identified compounds were used to assess their potential contributions to the aroma of cheeses supplemented with polyphenols (T0.1 and T0.2) compared to control cheeses.

Considering the OIR value > 1 in these experimental trials were selected 17 key odour-active compounds (Table 3).

As indicated in Table 3, the OIR value for 2,3-butanedione (diacetyl) was higher in all experimental samples, suggesting that this compound could be a key contributor to the buttery and nutty aroma of Caciotta cheese aged for 21 days. Its intensity was greater in the control and T0.2 samples than in treatment T0.1. Acetoin also exhibited a higher OIR, ranging from 22 to 42 across all samples, contributing to the aromatic notes of butter and sour milk.

In the cheese samples from the control group, hexanoic acid ethyl ester was identified as the key compound, with an OIR of 20, indicating its significant contribution to the cheese’s characteristic fruity note. Other important compounds that contributed to the overall aroma of the control group cheese included pentanal, heptanal, δ-decalactone, 2-decenal, 1-nonanol, nonanal, 2-nonenal, 2-nonanone, octanal, and 2-heptanone, all of which had OIR values ranging from 1.6 to 14.

The cheese made from cow milk fed with a lower level of polyphenols (T0.1) had higher OIRs for nonanal, 2-nonenal, and pentanal, indicating that these compounds were likely important for the aroma of this product. In contrast, the T0.2 cheese exhibited an OIR greater than 17 for 2-decenal, suggesting that this compound could significantly contribute to the aroma of the T0.2 group.

Finally, the key compounds in the T0.2 samples included diacetyl, hexanoic acid ethyl ester, acetoin, 2-decenal, pentanal, δ-decalactone, and heptanal, with OIR values ranging from 4.8 to 290.

### 2.5. Principal Component Analysis

Principal component analysis (PCA) is a statistical technique that reduces the dimensionality of data by transforming multiple variables into principal components. It is widely regarded as a key dimensionality reduction method. In the PCA model, the key volatile compounds with OIR > 1 and significant differences (*p* ≤ 0.05) were included as variables to determine their contribution to the odour profiles.

**Figure 2:**
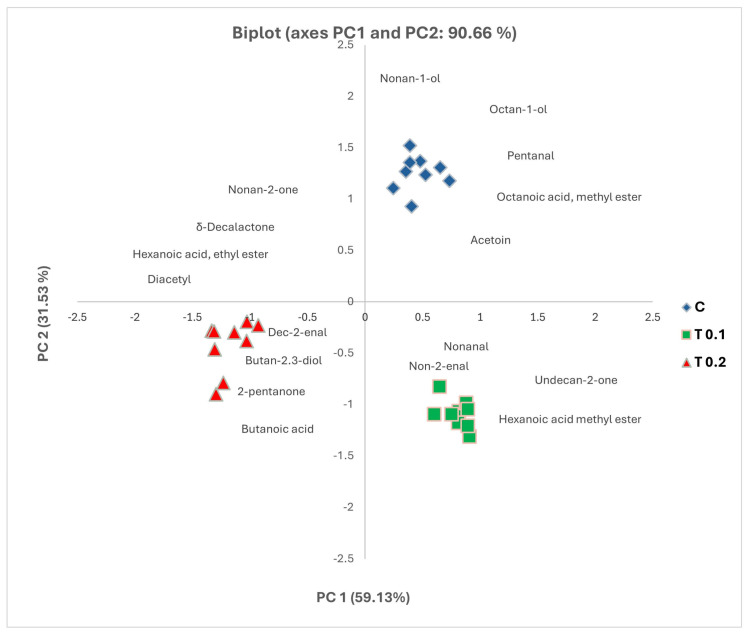
Principal component analysis (PCA) applied to key volatile compounds detected in Caciotta cheese from milk cows fed with different levels of spray-dried phenols. Scores and loading of the first two principal components. C = Control diet, T0.1 = diet with polyphenols at 0.1% of dry matter, T0.2 diet with polyphenols at 0.2% of dry matter.

As shown in Figure 2, the first two components (PC1 and PC2) explained 90.66% of the variance, with PC 1 and PC 2 explaining 59.13% and 31.53% of the variation, respectively.

The score plot of PC1 vs. PC2 shows three well-separated groups corresponding to the different dietary treatments (Figure 2), indicating that the differences in key odorant active content among the samples are evident.

The cheese samples from the C and T 0.1 groups are in the upper and lower right quadrants of the plot, respectively, indicating a clear separation from the other samples, as shown in Figure 1. In contrast, the cheese made from the milk of cows supplemented with a high level of polyphenols (0.2% of dry matter) clusters in the lower left quadrant. This suggests that the examined cheeses have different volatile profiles, indicating that the inclusion of polyphenols in the diet affects the distinct odour patterns in the cheese.

The key volatile compounds that contributed significantly to the variation in the PCA included 2-undecanone and octanoic acid methyl ester, which had positive loadings, while 2-decenal, hexanoic acid ethyl ester, δ-decalactone, 2,3-butanedione, and 2-nonanone had negative loadings for the first principal component (PC1). The second principal component (PC2) captured the variance associated with positive loadings from acetoin, pentanal, 1-octanol, and 1-nonanol, while negative loadings were identified for nonanal, 2-pentanone, hexanoic acid methyl ester, 2-nonenal, and butanoic acid.

In comparing the PCA score plots with the PCA loading plots, we observe that the C samples exhibited higher levels of 1-octanol, pentanal, octanoic acid methyl ester, and acetoin. In contrast, the cheese samples from the T0.1 group showed elevated levels of nonanal, 2-nonenal, undecanal, and hexanoic acid methyl ester. Additionally, the T0.2 cheese samples were closely associated with higher content of dec-2-enal, butanoic acid, butane-2,3-diol, and 2-pentanone. These findings are consistent with the data presented in Table 1. The PCA clearly demonstrates that polyphenol supplementation in cow diets significantly alters the volatile compound profile of cheese, leading to distinct odour characteristics and supporting the hypothesis that dietary treatments can modulate sensory properties in dairy products.

### 2.6. E-Nose Analysis

**Figure 3:**
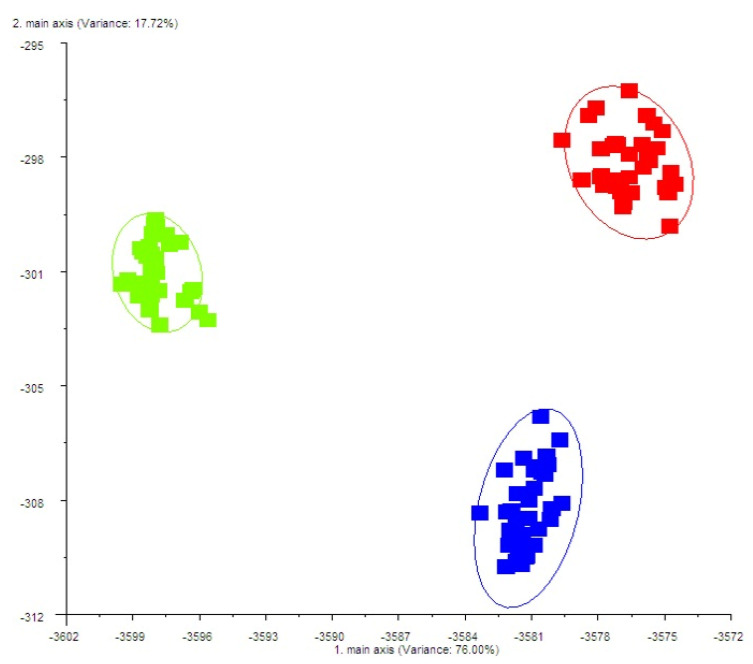
Linear discriminant analysis (LDA) of Caciotta cheese from milk cows fed with concentrate (C; blue data points), supplemented phenols at 0.1% of dry matter (T0.1; green data point), and supplemented phenols at 0.2% of dry matter (T0.2; red data point).

The electronic nose output was analyzed using linear discriminant analysis (LDA) to assess its effectiveness in distinguishing cheese odour profiles according to dietary treatment (Figure 3).

The LDA model explained 93.72% of the total variance in the dataset, indicating a strong discriminatory power. The results also showed that the LDA algorithm successfully classified the samples into three olfactory fingerprints, each associated with a specific dietary treatment.

This suggests that the inclusion of polyphenols in the cows’ diet significantly influences the odour patterns of the resulting cheese. These findings reinforce the utility of electronic nose technology, combined with multivariate statistical analysis, as a reliable tool for monitoring and characterizing aroma variations in dairy products linked to dietary nutritional strategies.

### 2.7. Correlation Between Key Volatile Compounds and Intelligent Sensory Signals

Seventeen key odour-active compounds were identified in the headspace of both control and polyphenol-supplemented cheese samples (Table 3). These volatile organic compounds (VOCs) were statistically correlated with the response signals from the electronic nose sensors. Most of the VOCs detected across all experimental groups exhibited significant correlations with specific sensor outputs.

**Figure 4:**
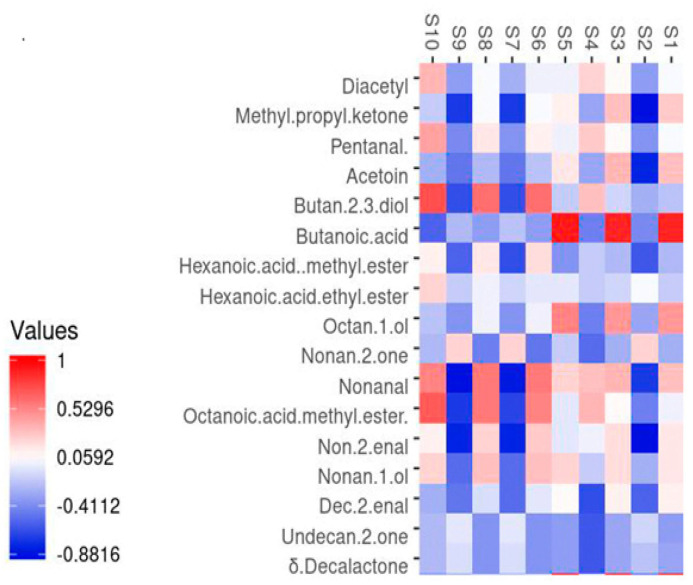
Key volatile compounds were plotted on the *Y* axis, while the *X* axis had the main sensors of the electronic nose. The hue of each tile on the heat map represents the degree of correlation for a specific sensor response and compound combination, as indicated by the colour key.

The heatmap presented in Figure 4 illustrates that the signal intensities from sensors S5 (W5C), S3 (W3C), and S1 (W1C) were positively correlated with butanoic acid. In contrast, sensors S10 (W3S), S8 (W2S), and S6 (W1S) showed positive correlations with 2,3-butanediol, octanoic acid methyl ester, and nonanal.

These sensor responses were consistent with the VOC pattern analysis, which revealed that cheese samples from the T0.2 treatment group were associated with higher concentrations of butanoic acid and 2,3-butanediol, as reported in Table 2.

Conversely, the signal intensities from sensors S2 (W5S), S7 (W1W), and S9 (W2W) were negatively correlated with several compounds, including methyl propyl ketone (2-pentanone), acetoin, 2,3-butanediol, hexanoic acid methyl ester, nonanal, (E)-2-nonenal, 1-nonanol, and (E)-2-decenal. Sensor S4 (W6S) exhibited negative correlations with δ-decalactone, 2-undecanone, (E)-2-decenal, and 2-nonanone.

The contributions of sensors S10 (W3S), S8 (W2S), and S6 (W1S) were particularly relevant for differentiating the T0.1 samples, as they were directly associated with the presence of nonanal.

Overall, many VOCs demonstrated measurable correlations with E-nose sensor responses, suggesting that the E-nose system is a reliable tool for distinguishing cheese samples based on their odour profiles and for rapidly monitoring the impact of polyphenol supplementation on cheese aroma.

## 3. Materials and Methods

### 3.1. Experimental Design

The experimental design was prepared in accordance with Directive 2010/63/EU of the European Parliament [39], which deals with the protection of animals used for scientific purposes.

The trial was performed on a commercial farm located in the southern region of Italy. Briefly, thirty Friesian cows were allotted into 3 groups homogeneous in terms of milk production per day (21.1 ± 1.6 L) and days in milk (29 ± 1 day) but receiving different diets.

The experimental trial lasted 4 weeks, taking place after 1 week of adaptation to the experimental diet. In the experimental period, the animals received three different diets: the control group (C) was fed with a pelleted concentrate, whereas the experimental group (T0.1) received pelleted concentrate supplemented with the polyphenols, recovered from oil mill wastewater by spray-drying, at 0.1% of dry matter basis to reach a total concentration of 480 mg/kg of phenolic compounds, and the T0.2 group received a concentrate supplemented with the phenol compounds at 0.2% of a dry matter to reach a total concentration of 960 mg/kg of phenolic compounds. All animals received polyphyte hay with a ratio of forage-concentrate of 60:40, and a formulated concentrate was offered daily for a total of 10.3 kg/head of dry matter. All concentrate ingredients and dry-sprayed phenols were incorporated into the pellets. All dairy cows consumed the whole daily amount of feed supplied because of the fixed amount of diet provided. The ingredients and chemical composition of the experimental diets are reported in Table 4.

### 3.2. Chemical Analysis of Feed and Cheese

The diet samples, relative to the dietary treatments, were collected daily, pooled weekly, and stored at −20 °C until further analysis. Table 3 shows the chemical composition of experimental diets and spray-dried olive oil mill wastewater. According to AOAC [40] methods, the dry matter (DM), ether extract (EE), and ash contents of the diets were calculated. The Van Soest et al. [41] procedure was used to assess the fibre fractions, including NDF, ADF, and ADL. The analysis of the fatty acid composition of dietary treatments was carried out using the method of Gray et al. [42]. Dry matter, protein, fat, and ash content in cheese were determined according to AOAC methods [40].

### 3.3. Spray-Dried Olive Mill Wastewater Phenols Analysis

This research is part of a broader project aimed at assessing the impact of diets enriched with varying concentrations of phenolic compounds extracted from olive mill wastewater on the quality of milk and its derived dairy products. Details on the phenol extraction from olive oil mill wastewater and analysis of spray-dried phenol compounds have been reported in a previous study [5,6].

### 3.4. Cheese Making

Raw milk (sum of pm and am milking) for 3 consecutive weeks was separately collected from the three experimental groups. The batches of milk, for each experimental group and for each time, were refrigerated (4 °C) and promptly transported to the dairy technology laboratory of the Research Centre for Animal Production and Aquaculture. A total of 3 cheesemaking sessions were carried out using the bulk milk for each group according to the following manufacturing protocol.

Bulk milk was pasteurized at 75 °C for 20 s, then cooled to 36 ± 1 °C. A commercial starter culture (Lyofast Y 082 D, Sacco SRL, Cadorago, Italy), containing *Streptococcus thermophilus* and *Lactobacillus delbrueckii* subsp. *bulgaricus*, was added at a concentration of 1.0 UC/100 L. After 30 min of incubation, calf rennet (75% chymosin, 25% pepsin; 1:18,000 strength; Caglificio Clerici, Cadorago, Italy) was added at 15 g/100 kg. Coagulation began after a further 30 min.

The curd was cut into hazelnut-sized pieces, portioned into 1 kg aliquots, placed in plastic moulds, and held at 50 °C until the pH reached 5.20 ± 0.1. Cheese was then salted in a 20% NaCl brine solution (1 h/kg of cheese) and transferred to a ripening room maintained at 10 ± 0.5 °C and 85% relative humidity.

After 21 days of ripening, 27 samples of Caciotta cheese were vacuum-packed and frozen at −80 °C until analysis.

### 3.5. Headspace-Solid Phase Microextraction-Gas Chromatography–Mass Spectrometry

Volatile compounds were extracted from cheese samples using solid-phase microextraction (SPME) and subsequently separated and identified by gas chromatography coupled with mass spectrometry (GC-MS).

Three grams of grated cheese samples were placed into glass vials (20 mL, Supelco, Bellefonte, PA, USA), mixed with 2.5 g of NaCl and added with 10 µL of internal standard solution (4-methyl 2-heptanone; 10 mg/kg in ethanol). The vials sealed with a polytetrafluoroethylene-silicone septum (Supelco, Bellefonte, PA, USA), were equilibrated for 30 min at 40 °C and stirred at 600 rpm. The adsorption phase was performed by exposing the preconditioned SPME fibre (50/30 μm DVB/CAR/PDMS, 2 cm; Supelco, Bellefonte, PA, USA) into the headspace of vials and magnetically stirring the samples at 600 rpm for 30 min at 40 °C. the Desorption of SPME fibre was performed, in splitless mode, in the gas chromatograph injector at 260 C for 5 min. Volatile organic compounds were separated by gas chromatography using an HP5 column (30 m 0.25 mm 0.25 mm; Agilent Technologies Co., Ltd., CA, USA). Thermal desorption of a SPME fibre was carried out in splitless mode, into gas chromatograph injector at 260 C for 5 min Gas chromatography analysis was performed on GC (model 6890 N, Agilent Technologies, Palo Alto, CA, USA) connected to a quadrupole mass selective detector (MSD model 5973, Agilent Technologies) operating in an electron ionization mode (internal ionization source was set at 230 °C; 70 eV) with a scan range from 29 to 400 *m*/*z*.

Separation was achieved on fused silica capillary column coated with dimethylpolysiloxane (HP1, 30 m, 0.32 mm inner diameter, 0.25 μm film thickness Agilent Technologies, Palo Alto, CA, USA). The gas chromatograph was operated using the following conditions: helium flow rate of 1 mL min^−1^; transfer line to MS 250 °C; interface: open splitless; temperature programme: 10 min at 40 °C; heating rate 4 °C min^−1^ to 240 °C and then held for 3 min.

An auto-tune of the GC-MS was carried out before the analysis to ensure optimal GC-MS performance. Each sample was analyzed in duplicate. The volatile compounds (VOCs) were identified by comparing the mass spectra with those in Wiley 8 (Wiley and Sons, Berlin, Germany) and NIST10 (National Institute of Standards and Technology US, Government library) library and using the AMDIS 7 peak deconvolution software. The results were expressed as a percentage of the total area of identified VOCs (Table 2).

### 3.6. E-Nose Analysis

The odour fingerprint analysis on cheese was evaluated using an electronic nose (PEN3, AIRSENSE Analytics GmbH, Schwerin, Germany). Ten metal oxide semiconductors with different chemical compositions and thicknesses formed a sensor array system (MOS) to provide selectivity toward volatile compound classes. A total of 10 g of cheese sample mL was placed in a 50 mL headspace bottle and kept at 30 °C for 60 min before detection. The flushing time was 300 s, the acquisition time was 60 s, and the sampling interval was 10 s. Data storage and multivariate statistical processing were performed using the workstation software Winmuster 1.6.2.18. Each sample was repeated eight times, and the data with stable measurement results were selected for statistical analysis. The performance description and the gas sensitivity range of the 10 metal oxide sensors are shown in Table 5.

### 3.7. Calculation of Odour Impact Ratio Values (OIRs)

The odour impact ratios (OIRs) indicate the extent to which volatile organic compounds (VOCs) contribute to the characteristic flavour of cheese. To quantify the odour impact of each volatile compound in Caciotta cheese samples, the OIR was calculated according to the method described by Santamarina-Garcia et al. [43], using the following equation: OIR = volatile relative abundance divided by odour threshold (μg/L or μg/kg).

However, determining odour impact ratios (OIRs) is complicated by variability in sensory threshold values, which depend on factors such as sample matrix, pH, temperature, and analytical methods. Ideally, thresholds should be established specifically for cheese. Due to limited available data, this study used threshold values of milk as reported in the literature [44]. According to Santamarina-Garcia et al. [43], an OIR greater than 1 indicates that a compound makes a perceptible contribution to the overall aroma, and higher values reflect stronger individual contributions.

### 3.8. Statistical Analysis

The data were analyzed using analysis of variance (SAS version 9.3, SAS Institute, Cary, NC, USA), with dietary treatment as a factor and sampling time as a repeated measure. Fisher’s LSD test was used to compare mean values. Standard PCA was applied to the volatile compounds composition of cheese samples to assess the relationship between the different variables and identify the most relevant factors of variability. The data acquired from the sensor array of the electronic nose was analyzed by linear discriminant analysis (LDA).

## 4. Conclusions

Incorporating spray-dried phenolic compounds recovered from olive oil mill wastewater into dairy cow diets can markedly influence the volatile compound profile of cheese, enhancing or modifying its aroma. Dietary supplementation with polyphenols notably affects the aroma profile of Caciotta cheese by changing the concentration of key VOCs. The variations in odour impact ratios across dietary treatments highlight the potential of nutritional strategies to enhance or tailor cheese flavour characteristics. Further sensory evaluations, including both panel and consumer tests, are required to validate these findings. The results obtained using the electronic nose were consistent with those from solid-phase microextraction gas chromatography–mass spectrometry, demonstrating that both analytical methods are effective for detecting odour changes in dairy products linked to dietary treatments. This also highlights the importance of integrating multiple analytical approaches to comprehensively characterize VOCs in cheese, thereby providing deeper insights into aroma differences among samples derived from varied feeding systems. Finally, the inclusion of olive mill wastewater by-products in dairy cow diets offers a viable strategy for sustainable agriculture, to valorise waste, reduce environmental impact, and advance circular economy principles by converting a pollutant into a valuable feed additive.

## Figures and Tables

**Table 1:** Chemical composition (%) in Caciotta cheese from milk cows fed with different levels of spray-dried phenols.

	Diets ^1^				
	C	T0.1	T0.2	SEM ^2^	*p*
Dry matter (%)	47.465	47.506	47.540	0.044	0.498
Protein (%)	20.527	20.861	20.887	0.038	0.839
Fat (%)	21.955	21.853	21.962	0.042	0.207
Ash (%)	2.460	2.442	2.526	0.089	0.788

^1^ C = Control diet, T0.1 = diet with phenols at 0.1% of dry matter, and T0.2 = diet with phenols at 0.2% of dry matter. ^2^ SEM = standard error of the means.

**Table 2:** Volatile compounds (%) in Caciotta cheese from milk cows fed with different levels of spray-dried phenols.

		Diets ^1^				
		C	T0.1	T0.2	SEM ^2^	*p*
**R.T. ^3^**						
1.467	Ethanol	0.807 ^b^	1.019 ^ab^	1.372 ^a^	0.098	0.018
1.547	Acetone	0.436 ^B^	1.094 ^A^	1.130 ^A^	0.038	0.0001
2.107	2,3-Butanedione	2.366 ^B^	1.641 ^B^	4.062 ^A^	0.192	0.0003
2.429	Acetic acid	13.508	9.689	12.175	1.093	ns
3.067	2-Pentanone	0.688 ^b^	1.110 ^ab^	1.570 ^a^	0.161	0.024
3.123	Pentanal	1.771 ^A^	1.332 ^B^	1.015 ^B^	0.081	0.0018
3.418	2-Butanone, 3-hydroxy	5.933 ^A^	4.359 ^B^	3.119 ^C^	0.277	0.0011
5.47	Cyclobutanol	0.597 ^A^	0.097 ^B^	0.082 ^B^	0.025	0.0001
5.827	Octane	0.729	0.828	0.612	0.066	ns
5.949	Hexanal	0.759	0.583	0.762	0.075	ns
6.698	2,3-Butanediol	0.786 ^B^	1.004 ^B^	1.825 ^A^	0.134	0.0036
8.567	Butanoic acid	13.460 ^B^	19.339 ^A^	19.120 ^A^	0.708	0.0017
10.041	2-Heptanone	0.813	0.997	0.800	0.099	ns
10.381	Heptanal	0.743	0.704	0.579	0.054	ns
11.451	Hexanoic acid, methyl ester	0.090 ^B^	0.422 ^A^	0.087 ^B^	0.039	0.0013
14.237	Hexanoic acid, ethyl ester	1.535 ^B^	0.716 ^C^	2.939 ^A^	0.079	0.0001
14.445	Octanal	0.785	0.911	1.235	0.195	ns
15.627	Hexanoic acid	19.633	20.184	19.658	0.791	ns
16.919	1-Octanol	0.783 ^A^	0.482 ^B^	0.311 ^B^	0.052	0.0020
17.585	2-Nonanone	1.151 ^A^	0.685 ^B^	1.289 ^A^	0.068	0.0019
17.956	Nonanal	0.533 ^B^	3.814 ^A^	0.488 ^B^	0.126	0.0001
18.522	Octanoic acid, methyl ester	0.776 ^A^	0.680 ^A^	0.060 ^B^	0.049	0.0001
19.777	2-Nonenal	0.107 ^B^	0.727 ^A^	0.127 ^B^	0.094	0.0054
20.279	1-Nonanol	0.290 ^a^	0.058 ^b^	0.131 ^ab^	0.038	0.0130
20.851	Octanoic acid, ethyl ester	0.174 ^B^	0.064 ^B^	0.550 ^A^	0.039	0.0001
21.234	Octanoic Acid	6.162	5.180	6.858	0.478	ns
22.797	2-Decenal	0.359 ^B^	0.207 ^B^	1.568 ^A^	0.053	0.0001
23.485	Nonanoic acid	0.296 ^B^	0.146 ^C^	0.647 ^A^	0.024	0.0001
23.672	2-Undecanone	0.575 ^B^	0.986 ^A^	0.139 ^C^	0.012	<0.0001
26.605	Decanoic acid	2.267 ^B^	2.014 ^B^	2.874 ^A^	0.062	0.0002
26.75	Dodecanal	0.448 ^A^	0.214 ^B^	0.248 ^B^	0.020	0.0004
28.462	1-Dodecanol	0.347 ^ab^	0.223 ^b^	0.424 ^a^	0.043	0.046
28.943	Hexadecane	0.266 ^ab^	0.145 ^b^	0.315 ^a^	0.032	0.026
29.155	δ-Decalactone	0.304 ^B^	0.207 ^C^	0.442 ^A^	0.012	0.0001
29.739	Nonadecane	0.067 ^B^	0.040 ^C^	0.214 ^A^	0.006	<0.0001
31.063	Dodecanoic acid	1.134 ^A^	0.517 ^B^	0.948 ^A^	0.069	0.002
31.315	Tridecane	0.184 ^A^	0.085 ^B^	0.079 ^B^	0.006	0.0001
31.449	Diethyl phthalate	0.132 ^ab^	0.156 ^a^	0.077 ^b^	0.014	0.0208
31.703	Pentadecanal	0.144 ^b^	0.178 ^ab^	0.214 ^a^	0.014	0.0385
34.053	δ-Nonalactone	0.352 ^a^	0.088 ^b^	0.217 ^ab^	0.038	0.0078
35.398	Tetradecanoic acid	2.654 ^A^	1.485 ^B^	2.256 ^A^	0.095	0.0004
39.408	Hexadecanoic acid	12.488 ^b^	14.244 ^ab^	15.558 ^a^	0.520	0.016
41.272	1-Octadecanol	1.373 ^B^	0.353 ^C^	2.335 ^A^	0.049	0.0001
42.689	Oleic Acid	0.513	0.477	0.516	0.029	ns
43.144	Octadecanoic acid	0.682	0.516	0.518	0.117	ns

Means in the same row with different letters indicate significant differences (a, b = *p* < 0.05; A, B, C = *p* < 0.0001). ^1^ C = Control diet, T0.1 = diet with phenols at 0.1% of dry matter, and T0.2 = diet with phenols at 0.2% of dry matter. ^2^ SEM = standard error of the means; ^3^ R.T. = Retention Time.

**Table 3:** Odour Impact Ratio value (OIR) and sensory description of key volatile compounds detected in Caciotta cheese from milk cows fed with different levels of spray-dried phenols.

	Odour Threshold	OIR			Odour Descriptors
	(μg/kg)	C ^1^	T0.1	T0.2	
2-Pentanone	0.5	1.376	2.220	3.140	Sweety, fruity
Pentanal	0.13	13.623	10.246	7.808	Chemical. pungent
2-Butanone 3-hydroxy	0.14	42.379	31.136	22.279	Butter like. Sour milk
2,3-Butanediol	1	0.786	1.004	1.825	Buttery, creamy
Butanoic acid	18	0.748	1.074	1.062	Strong cheese unpleasant
Hexanoic acid. methyl ester	0.075	1.200	5.627	1.160	Fruity, cheese
1-Octanol	0.5	1.566	0.964	0.622	Fat, metal
2-Nonanone	0.5	2.302	1.370	2.578	Fruity pleasant
Nonanal	0.22	2.423	17.336	2.218	Tallow, animal
Octanoic acid. methyl ester	0.5	1.552	1.360	0.120	Wax sweet
2-Nonenal	0.045	2.378	16.156	2.822	Green
1-Nonanol	0.08	3.625	0.725	1.638	citrus
2-Decenal. (E)-	0.092	3.902	2.250	17.043	Pungent
2-Undecanone	0.5	1.150	1.972	0.278	Floreal
δ-Decalactone	0.064	4.750	3.234	6.906	Peachy, coconut

^1^ C = Control diet, T0.1 = diet with phenols at 0.1% of dry matter, and T0.2 = diet with phenols at 0.2% of dry matter.

**Table 4:** Ingredients (% as fed basis) chemical composition (g/100 g), fatty acids (g/100 g of total fat) and polyphenolic compounds of diets offered.

	Diets ^1^		
	C	T0.1	T0.2
Polyphite Hay	60	60	60
Maize grain	27	27	27
Wheat bran	16.2	16.1	16
Hulled soybean flour	14	14	14
Maize dried distillers’ grain	14	14	14
Distiller’s Dried Grains with Soluble	14	14	14
Hulled sunflower seed flour	13.6	13.6	13.6
Barley grain	0.2	0.2	0.2
Molasses	0.2	0.2	0.2
Dried beet pulp	0.2	0.2	0.2
Spray-dried olive mill wastewater phenolics	-	0.1	0.2
Vitamin-mineral supplement	0.6	0.6	0.6
**Chemical Composition**			
Dry matter	88.827	88.829	88.831
Crude protein	15.71	15.70	15.70
Ether extract	3.00	3.00	3.00
Fibre	27.01	27.00	27.00
Ash	7.293	7.295	7.297
NDF	51.20	51.19	51.17
ADF	6.03	6.02	6.02
ADL	4.44	4.43	4.43
UFL (Kg/S.S.)	0.84	0.84	0.84
Fatty acids (g/100 g of fatty acids)			
C16:0	11.64	11.64	11.64
C18:0	21.69	21.69	21.69
C18:1 n-9	10.53	10.55	10.56
C18:2 n-6	28.15	28.13	28.12
C18:3 n-3	7.00	7.00	7.00
Others	20.98	20.98	20.98

^1^ C = Control diet, T0.1 = diet with polyphenols 0.1% on dry matter, T0.2 diet with polyphenols 0.2% on dry matter.

**Table 5:** Sensor sensitivities and detection limits for the PEN3 sensor array.

Sensor Number	Sensor Name ^1^	Sensor Sensitives	Detection Limits
1	W1C	Aromatic organic compounds	Toluene, 10 mg kg^−1^
2	W5S	Very sensitive, broad range sensitivity, reacts to nitrogen oxides, very sensitive with negative signal	NO_2_, 1 mg kg^−1^
3	W3C	Ammonia, also used as sensor for aromatic compounds	Benzene, 10 mg kg^−1^
4	W6S	Detects mainly hydrogen gas	H_2_, 0.1 mg kg^−1^
5	W5C	Alkanes, aromatic compounds, and nonpolar organic compounds	Propane, 1 mg kg^−1^
6	W1S	Sensitive to methane, broad range of organic compounds detected	CH_3_, 100 mg kg^−1^
7	W1W	Detects inorganic sulphur compounds, e.g., H_2_S. Also sensitive to many terpenes and sulfur-containing organic compounds	H_2_S, 1 mg kg^−1^
8	W2S	Detects alcohol, partially sensitive to aromatic compounds, broad range	CO, 100 mg kg^−1^
9	W2W	Aromatic compounds, inorganic sulphur and organic compounds	H_2_S, 1 mg kg^−1^
10	W3S	Reacts to high concentrations (>100 mg kg^−1^) of methane and aliphatic organic compounds	Not determined

^1^ As reported in the ‘‘sensors options’’ of the e-nose software (Winmuster 1.6.2.5, Airsense Analytics GmbH, Schwerin, Germany.

## Data Availability

The authors confirm that the data supporting the findings of this study are available on request.
